# Supplementary material for: Structural, in silico, and functional analysis of a Disabled-2-derived peptide for recognition of sulfatides
Source: Sci Rep. 2020 Aug 11;10:13520. doi: 10.1038/s41598-020-70478-0 (PMC7421900; doi:10.1038/s41598-020-70478-0)
Supplement: Supplementary file 1 — Supplementary Information. [file 41598_2020_70478_MOESM1_ESM.pdf]

SUPPLEMENTARY INFORMATION

Structural, *in silico*, and functional analysis of Disabled-2-derived peptide for recognition to sulfatides

Wei Song [1], Carter J. Gottschalk [2], Tuo-Xian Tang [1], Andrew Biscardi [1], Jeffrey F. Ellena [3]

Carla V. Finkielstein [4], Anne M. Brown [2], and Daniel G. S. Capelluto [1,*]

[1] Protein Signaling Domains Laboratory, Department of Biological Sciences, Fralin Life Sciences Institute, and Center for Soft Matter and Biological Physics, Virginia Tech, Blacksburg, VA 24061, United States; [2] Research and Informatics, University Libraries, Biochemistry Department, and Center for Drug Discovery, Virginia Tech, Blacksburg, VA, United States; [3] Biomolecular Magnetic Resonance Facility, University of Virginia, Charlottesville VA, 22904, United States; [4] Integrated Cellular Responses Laboratory, Fralin Biomedical Research Institute, Department of Biological Sciences, and Center for Drug Discovery, Virginia Tech, Roanoke, VA 24016, United States.

[*] Corresponding author: Tel: + 540 231-0974; E-mail address: capellut@vt.edu

Material and Methods

Circular dichroism

Far-UV (190-250 nm) circular dichroism (CD) spectra were collected using a Jasco PFD-425 S temperature control unit-equipped J-815 spectropolarimeter at 10 µM Dab2 SBP constructs in 10 mM citrate (pH 5), 40 mM KF and in the presence of 10 mM DPC. Five CD spectral accumulations were collected at a 1-nm bandwidth with a response time of 1 s and at a scan speed of 20 nm/min at 23°C. Buffer spectra were also recorded under the same experimental conditions and subtracted from the peptide spectra.

Table S1. Residue-sulfatide interactions of the lowest energy pose of Dab2 SBP. Residue interactions were determined using Schrödinger-Maestro.

Dab2 SBP

Interaction Type	Backbone	Polar	Hydrophobic	Hydrogen Bond Acceptor	Hydrogen Bond Donor	Aromatic	Charged Residue
Residues	K30<br>P32<br>E33<br>K34<br>T35	E33<br>T35<br>D36<br>R42<br>K49<br>K51	Y38<br>L39<br>F43<br>Y50<br>L54<br>I55			Y38<br>F43<br>Y50	E33<br>D36<br>R42<br>K49<br>K51

Dab2 SBP R42A

Interaction Type	Backbone	Polar	Hydrophobic	Hydrogen Bond Acceptor	Hydrogen Bond Donor	Aromatic Residue	Charged Residue
Residue	K30<br>P32<br>E33<br>K34<br>T35	K29<br>E33<br>T35<br>K49<br>K53	P32<br>Y38<br>L39<br>A42<br>Y50<br>L54			Y38<br>Y50	K29<br>E33<br>K49<br>K53

Dab2 SBP R42K

Interaction Type	Backbone	Polar	Hydrophobic	Hydrogen Bond Acceptor	Hydrogen Bond Donor	Aromatic Residue	Charged Residue
Residue	K30<br>P32<br>E33<br>K34<br>Y50	E33<br>T35<br>K42<br>K49<br>K53	P32<br>Y38<br>L39<br>Y50<br>L54	Y38		Y38<br>Y50	E33<br>K42<br>K49<br>K53

Dab2 SBP Y38A

Interaction Type	Backbone	Polar	Hydrophobic	Hydrogen Bond Acceptor	Hydrogen Bond Donor	Aromatic Residue	Charged Residue
Residue	K30<br>G31<br>P32<br>K34<br>T35<br>A38<br>L39<br>L54	T35<br>R42<br>D46<br>K49	P32<br>A38<br>L39<br>Y50<br>L54	T35	R42	Y50	R42<br>D46<br>K49

Dab2 SBP K49A/K51A/K53A

Interaction Type	Backbone	Polar	Hydrophobic	Hydrogen Bond Acceptor	Hydrogen Bond Donor	Aromatic Residue	Charged Residue
Residue	K30<br>P32<br>E33<br>K34<br>T35<br>A49<br>Y50	K29<br>E33<br>T35<br>R42<br>D46	Y38<br>L39<br>A49<br>Y50<br>A53<br>L54	Y50		Y38<br>Y50	K29<br>E33<br>R42<br>D46

DAB2 SBP Y50A

Interaction Type	Backbone	Polar	Hydrophobic	Hydrogen Bond Acceptor	Hydrogen Bond Donor	Aromatic Residue	Charged Residue
Residue	K30<br>P32<br>E33<br>D46<br>G47<br>A50<br>K51<br>K53	K29<br>K30<br>E33<br>T35<br>R42<br>K51<br>K53	Y38<br>L39<br>A50<br>L54	K30		Y38	K29<br>K30<br>E33<br>R42<br>K51<br>K53

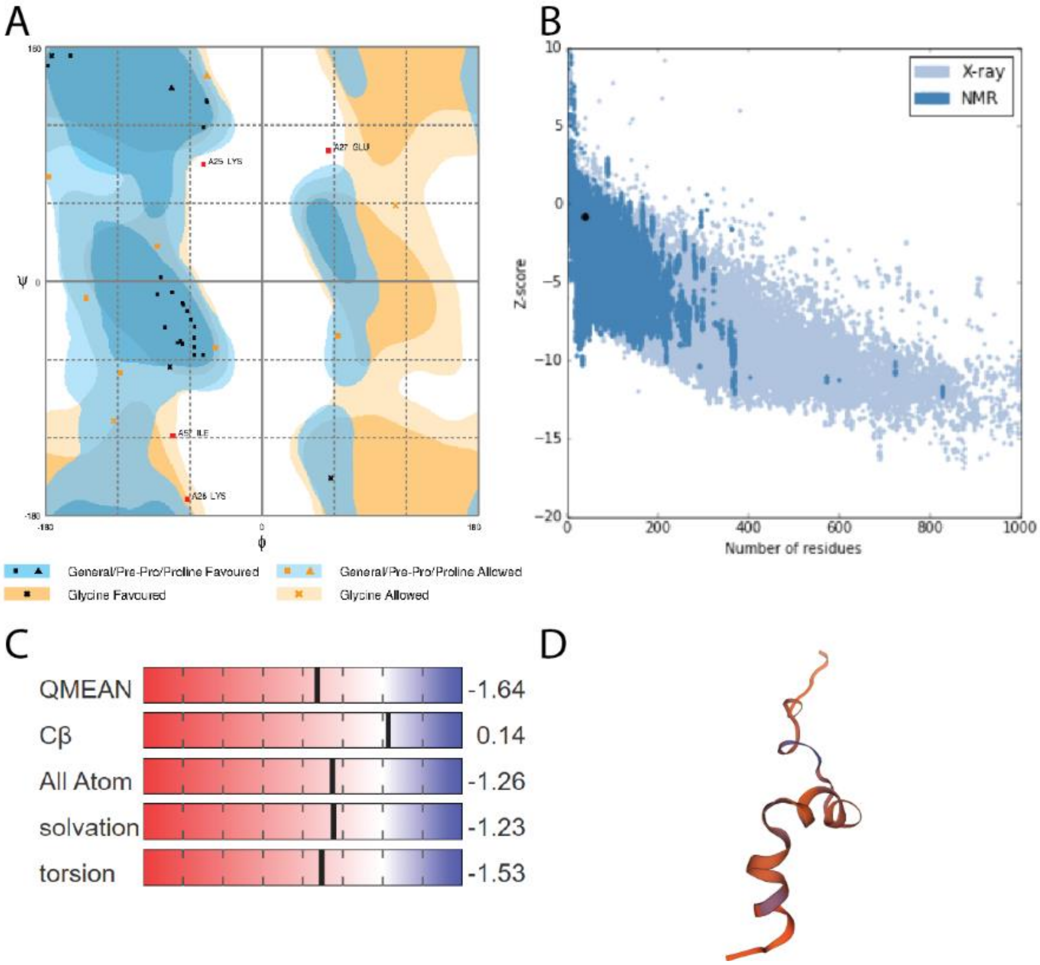

Fig. S1. Model validation of Dab2 SBP after energy minimization. (**A**) Ramachandran plot of (φ, ψ) angles for all residues represented as favorable and allowed (black and yellow squares, triangles, and Xs, 89.5%) and outlier (red squares, triangles, and Xs, 10.5%). (**B**) ProSA Z-Score represented as a black dot on the blue plot (-0.84) comparing structure to similar sequence length proteins resolved by NMR or x-ray crystallography. (**C**) Summary of QMEAN scores assessing structure quality based on solvation and torsion angle. (**D**) 3D-Model of QMEAN structure assessment scores mapped onto model.

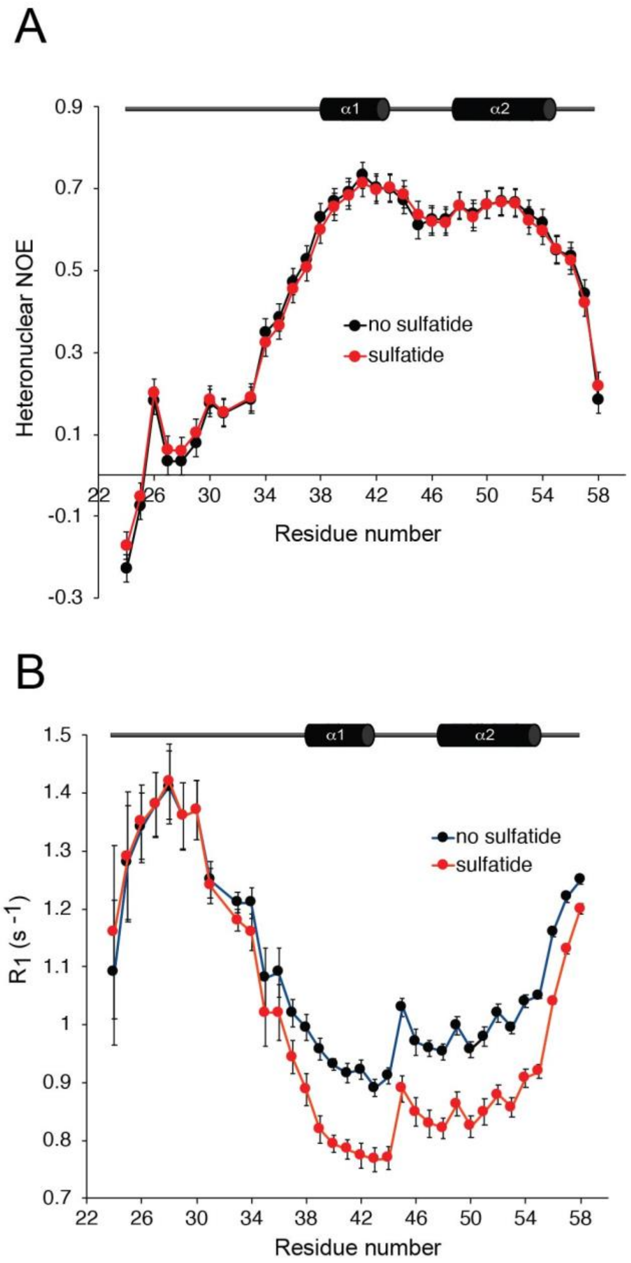

Fig. S2. Sulfatide-dependent Dab2 SBP dynamics characterized by NMR relaxation measurements. ^{1}H–^{15}N NOE ratio (**A**) and longitudinal relaxation rates, R_1 (**B**), of DPC-embedded Dab2 SBP in the absence (black) or presence (red) of 8-fold DPC-embedded sulfatides. The secondary structure of Dab2 SBP is depicted at the top of each panel.

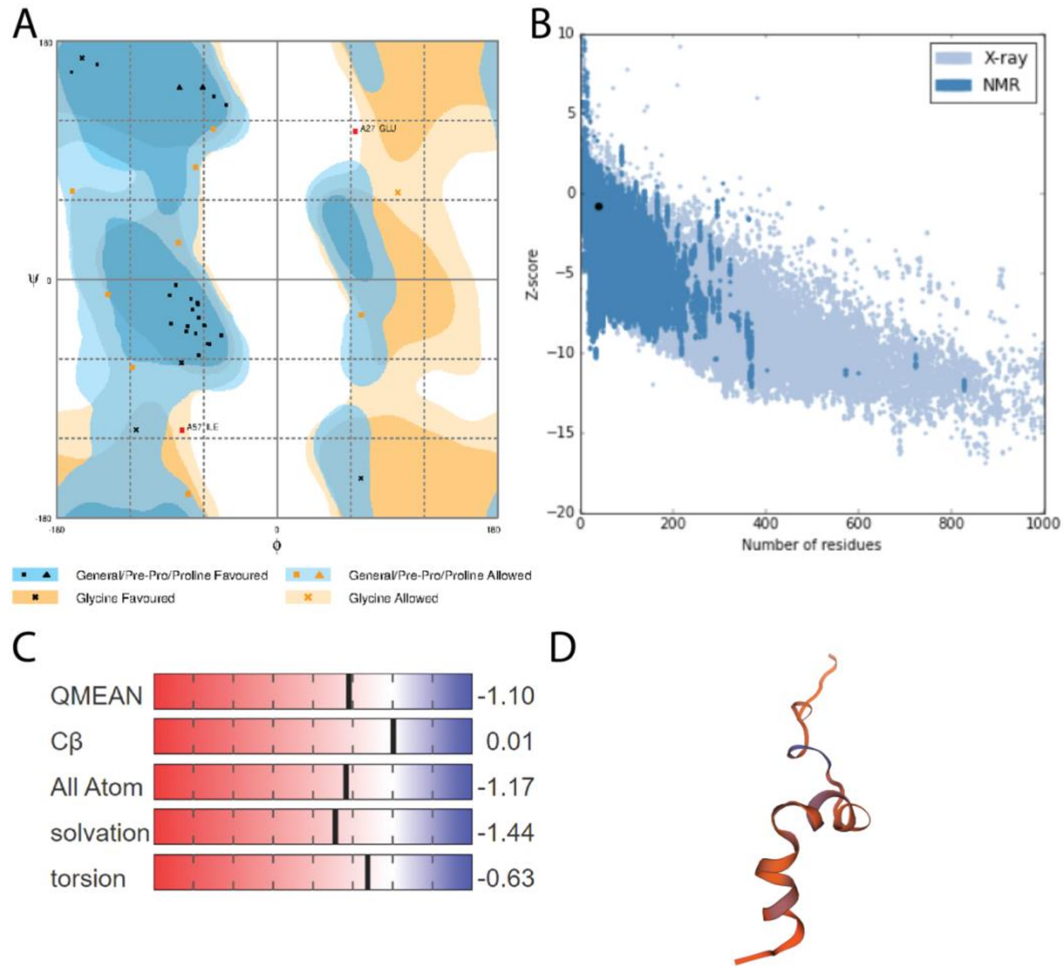

Fig. S3. Model validation of Dab2 SBP R42A after energy minimization. (**A**) Ramachandran plot of (φ, ψ) angles for all residues represented as favorable and allowed (black and yellow squares, triangles, and Xs, 94.7%) and outlier (red squares, triangles, and Xs, 5.3%). (**B**) ProSA Z-Score represented as a black dot on the blue plot (-0.8) comparing structure to similar sequence length proteins resolved by NMR or x-ray crystallography. (**C**) Summary of QMEAN scores assessing structure quality based on solvation and torsion angle. (**D**) 3D-Model of QMEAN structure assessment scores mapped onto model.

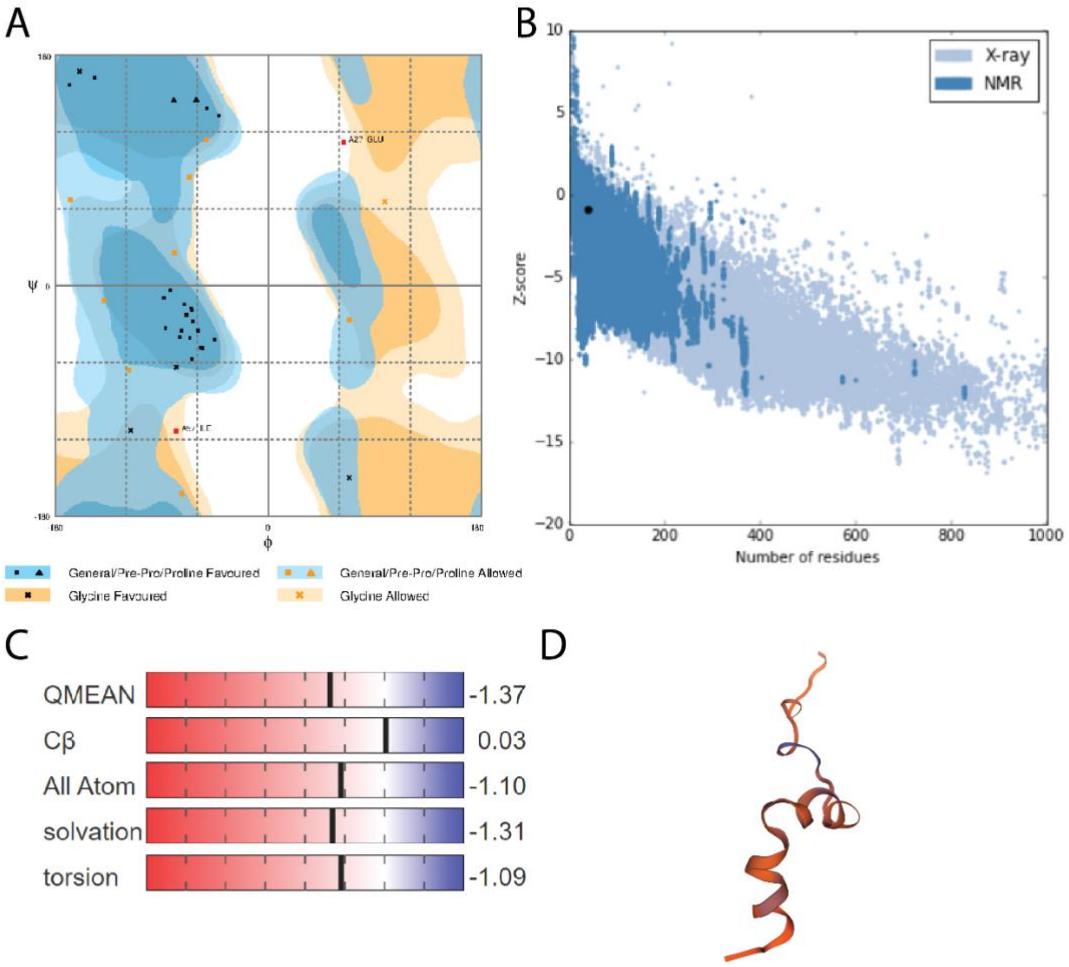

Fig. S4. Model validation of Dab2 SBP R42K structure after energy minimization. (**A)** Ramachandran plot of (φ, ψ) angles for all residues represented as favorable and allowed (black and yellow squares, triangles, and Xs, 94.7%) and outlier (red squares, triangles, and Xs, 5.3%). (**B**) ProSA Z-Score represented as a black dot on the blue plot (-0.9) comparing structure to similar sequence length proteins resolved by NMR or x-ray crystallography. (**C**) Summary of QMEAN scores assessing structure quality based on solvation and torsion angle. (**D**) 3D-Model of QMEAN structure assessment scores mapped onto model.

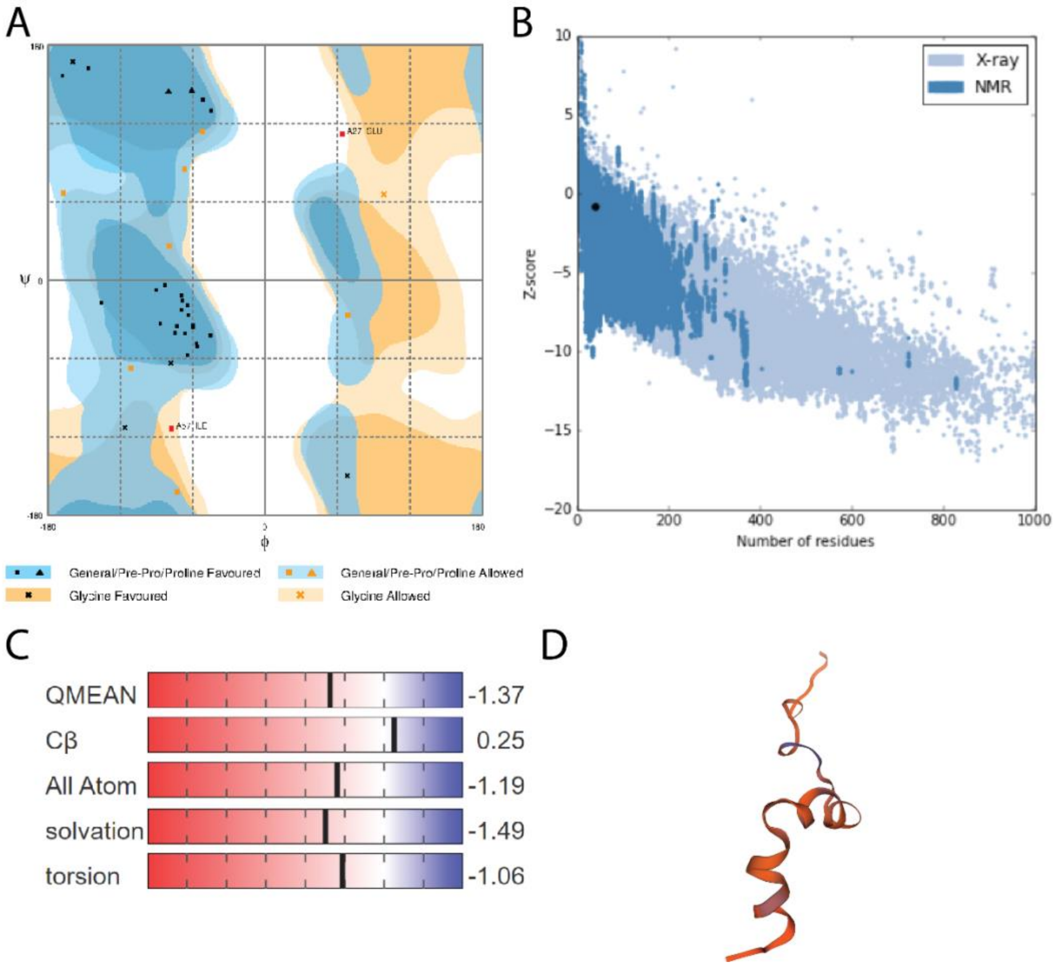

Fig. S5. Model validation of Dab2 SBP Y38A after energy minimization. (**A**) Ramachandran plot of (φ, ψ) angles for all residues represented as favorable and allowed (black and yellow squares, triangles, and Xs, 94.7%) and outlier (red squares, triangles, and Xs, 5.3%). (**B**) ProSA Z-Score represented as a black dot on the blue plot (-0.78) comparing structure to similar sequence length proteins resolved by NMR or x-ray crystallography. (**C**) Summary of QMEAN scores assessing structure quality based on solvation and torsion angle. (**D**) 3D-Model of QMEAN structure assessment scores mapped onto model.

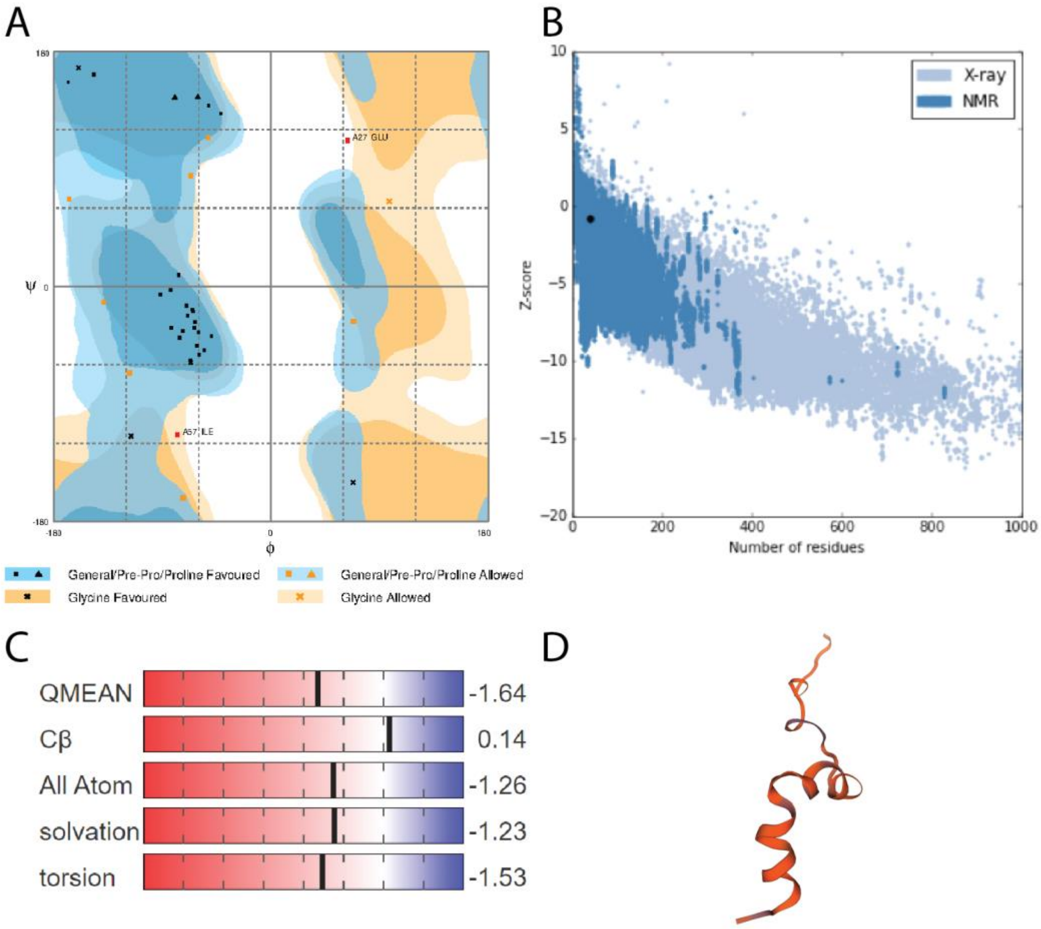

Fig. S6. Model validation of Dab2 SBP K49A/K51A/K53A Dab2 after energy minimization. (**A**) Ramachandran plot of (φ, ψ) angles for all residues represented as favorable and allowed (black and yellow squares, triangles, and Xs, 94.7%) and outlier (red squares, triangles, and Xs, 5.3%). (**B**) ProSA Z-Score represented as a black dot on the blue plot (-0.77) comparing structure to similar sequence length proteins resolved by NMR or x-ray crystallography. (**C**) Summary of QMEAN scores assessing structure quality based on solvation and torsion angle. (**D**) 3D-Model of QMEAN structure assessment scores mapped onto model.

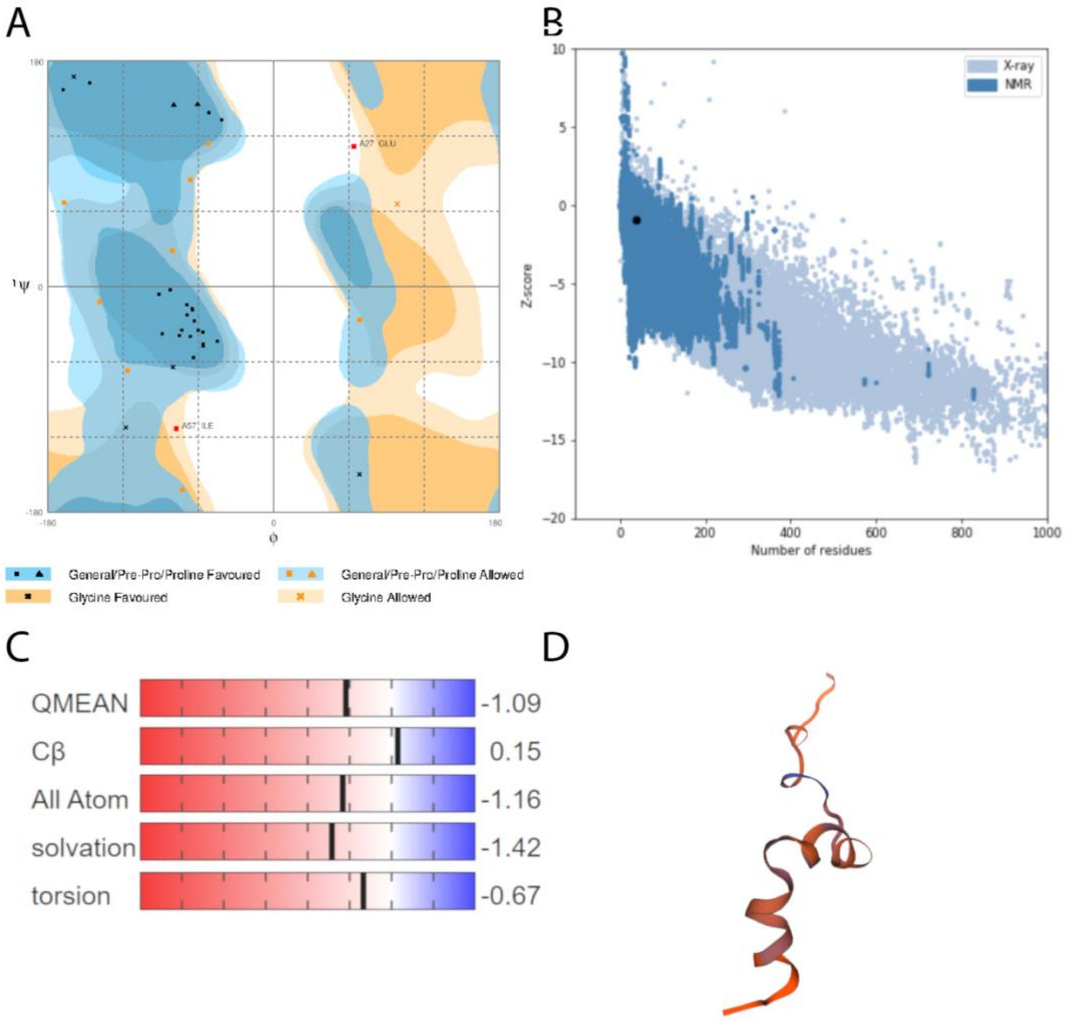

Fig. S7. Model validation of Dab2 SBP Y50A structure after energy minimization. **(A)** Ramachandran plot of (φ, ψ) angles for all residues represented as favorable and allowed (black and yellow squares, triangles, and Xs, 94.7%) and outlier (red squares, triangles, and Xs, 5.3%). **(B)** ProSA Z-Score represented as a black dot on the blue plot (-0.89) comparing structure to similar sequence length proteins resolved by NMR or x-ray crystallography. **(C)** Summary of QMEAN scores assessing structure quality based on solvation and torsion angle. **(D)** 3D-Model of QMEAN structure assessment scores mapped onto model.

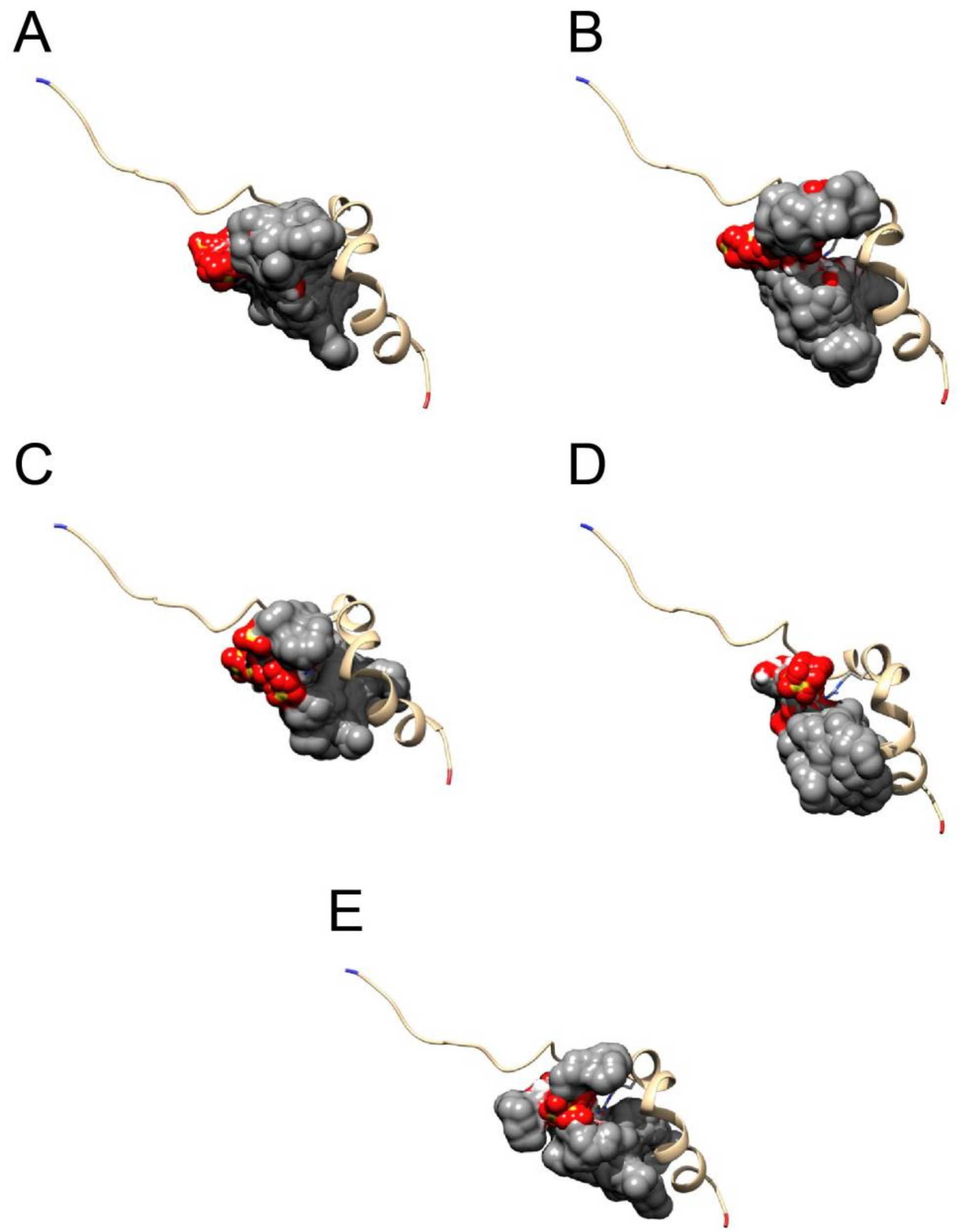

Fig. S8. Surface representation of all sulfatide poses docked to Dab2 SBP mutants. (**A**) Dab2 SBP R42A; (**B**) Dab2 SBP R42K; (**C**) Dab2 SBP Y38A; (**D**) Dab2 SBP K49A/K51A/K53A; (**E**) Dab2 SBP Y50A. Dab2 SBP mutant structures are rendered as cartoon colored tan with the N-terminus colored blue and C-terminus colored red. R42, when visible, is shown as stick colored gray and by atom type. Sulfatide head groups are colored red with their sulfo groups yellow, whereas the acyl chains are colored gray. The nine poses, produced by AutoDock Vina, are shown as surface colored gray and by atom type.

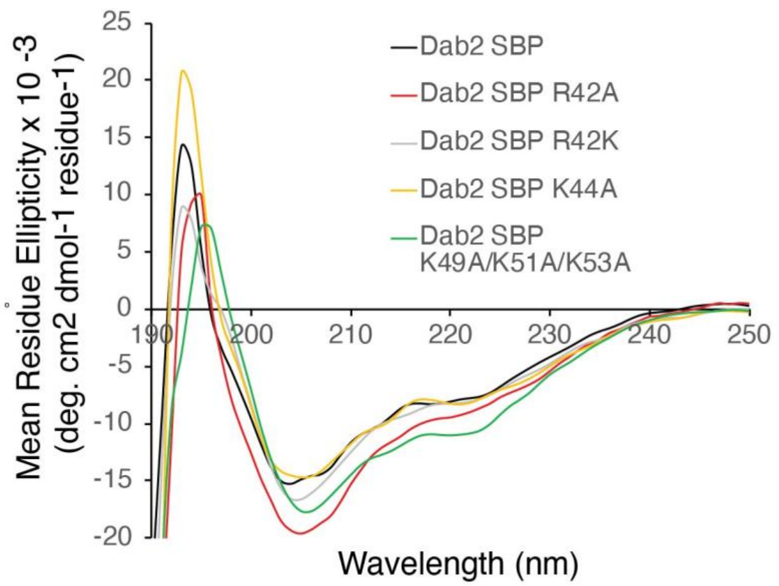

Fig. S9. Far-UV CD spectra of DPC-embedded Dab2 SBP (black), Dab2 SBP R42A (red), Dab2 SBP R42K (gray), Dab2 SBP K44A (orange), and Dab2 SBP K49A/K51A/K53A (green).
